# Size-matching in lung transplantation using the adjusted total lung capacity ratio

**DOI:** 10.1016/j.jhlto.2026.100595

**Published:** 2026-05-16

**Authors:** Iulia-Beatrice Sotrea, Julie Morisset, Maxime Tetu, Matthieu Glorion, Moishe Liberman, Pasquale Ferraro, Basil S. Nasir

**Affiliations:** a Division of Thoracic Surgery, Department of Surgery, Centre Hospitalier de l′Université de Montréal, Montréal, Québec, Canada; b Division of Pulmonology, Department of Medicine, Centre Hospitalier de l′Université de Montréal, Montreal, Quebec, Canada; c Department of Thoracic Surgery and Lung Transplantation, Foch Hospital, Suresnes, France

**Keywords:** lung transplantation, size matching, ajusted total lung capacity ratio, donor-recipient matching

## Abstract

**Background:**

Evidence guiding optimal size-matching in lung transplantation is limited, and no consensus exists. Most current approaches fail to account for the recipient’s underlying pathology. A recently proposed “adjusted” total lung capacity (TLC) ratio, incorporating actual (aTLC) and predicted TLC (pTLC), may offer a physiologically relevant size-matching strategy. However, its clinical performance has not been reported.

**Methods:**

We retrospectively reviewed 376 double lung transplants at our center between January 2017 and March 2023, excluding lobar transplants. We calculated the adjusted TLC ratio as the donor pTLC divided by the mean of the recipient pTLC and aTLC. Patients were categorized into 3 groups as follows: all recipients (Cohort A, n = 376), restrictive lung disease (Cohort B, n = 204), and restrictive disease without volume-reduction procedures (Cohort C, n = 150). The primary outcome was peak posttransplant forced expiratory volume in 1 s (FEV1). Secondary outcomes included baseline lung allograft dysfunction (BLAD), chronic lung allograft dysfunction (CLAD), survival, and time to normalization of lung function.

**Results:**

Based on posttransplant peak FEV1, we identified an adjusted TLC ratio of 0.9 to 1.0 as the optimal range for lung-size matching. Optimally sized (0.9-1.0) and undersized (0.79-0.89) grafts showed the highest FEV1 posttransplant. Oversized grafts (>1.0) were associated with lower FEV1 across all cohorts and higher BLAD incidence in Cohort A. The adjusted TLC ratio did not influence survival.

**Conclusion:**

Size-matching using an adjusted TLC ratio of 0.79 to 1.0 correlates with improved long-term outcomes in lung transplantation, whereas oversizing may impair lung function. The effect on survival remains inconclusive due to study limitations.

Matching donor lungs to recipients involves considering factors such as blood group, donor-specific antibodies, and size. The importance of size-matching on both short and long-term outcomes requires further definition.^1^, ^2^, ^3^ Current size-matching practices vary widely, with centers using height, weight, imaging studies, predicted total lung capacity (pTLC), or a combination thereof.^2^, ^3^, ^4^, ^5^ Acceptable matching standards are undefined, such as whether to match the recipient predicted TLC (pTLC) or actual TLC (aTLC) and the appropriate ratio. Additionally, there is no consensus on which outcome defines successful size-matching—early survival, long-term survival, lung function, or incidence of chronic lung allograft dysfunction (CLAD).^1^, ^2^, ^3^, ^4^, ^5^, ^6^ Finally, disease-specific recommendations differ, adding to the knowledge gaps in size-matching for lung transplants.^2^, ^3^, ^5^

Height-based matching overlooks sex-related lung size differences.^6^ Matching for pTLC resolves this. The International Society for Heart and Lung Transplantation (ISHLT) advises matching donor pTLC to 75% to 125% of the recipient’s pTLC.^7^ Riddell et al. recommends a donor-recipient pTLC ratio of 0.8 to 1.2.^8^ Neither approach accounts for the underlying lung disease, and the need for such consideration remains unclear. Barnard et al. proposed that in pulmonary fibrosis, optimal matching occurs when donor pTLC is within 15% to 20% above or below the calculated midpoint between the recipient pTLC and aTLC (Figure 1), whereas in emphysema, donor lungs should fall between 67% and 100% of the recipient's aTLC.^2^ The midpoint equation is noteworthy for incorporating the underlying restrictive physiology by including the recipient's aTLC. However, there are no reports on the performance of using this “adjusted” recipient TLC ratio.

**Figure 1:**
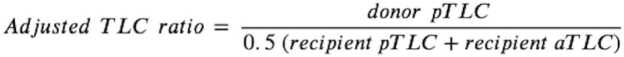
Adjusted TLC ratio

In this study, we aim to evaluate the performance of the adjusted TLC ratio in size-matching donor lungs to recipients.

## Methods

We reviewed all adult lung transplant recipients who underwent transplantation between January 2017 and March 2023 at the Centre Hospitalier de l′Université de Montreal (CHUM). We excluded patients who received lobar or single lung transplantation. We analyzed data on recipient and donor demographics, intraoperative course, and postoperative and long-term outcomes. Donor and recipient heights were recorded in centimeters; recipient height was measured by the nutritionist during the anthropometric evaluation, and donor height was obtained from organ procurement organization. In the earlier study period, size-matching at our center was primarily based on height, aiming to be within 7 cm. In addition, larger donor lungs were generally preferred for recipients with obstructive lung disease, and smaller lungs for those with restrictive lung disease. Beginning in mid-2019, our approach shifted to using pTLC donor-to-recipient ratio, with a preferred range of 75% to 125%, in line with previously published recommendations from the ISHLT.^7^

We calculated pTLC for donors and recipients using the Global Lung Function Initiative (GLI) calculators for Spirometry, TLCO, and Lung volume.^9^ The calculator incorporates sex, height, and age to generate pTLC values with defined upper and lower limits of normal.^10^ We obtained the recipient's aTLC from the most recent pre-transplant pulmonary function testing. We calculated the *adjusted TLC ratio*, as described by Barnard et al., by dividing the donor's pTLC by the midpoint between the recipient's pTLC and aTLC [dpTLC / 0.5(rpTLC + raTLC)].^2^

Although Barnard et al. described the formula for patients with pulmonary fibrosis, we evaluated its application across all lung transplant recipients. We first analyzed the entire cohort (Cohort A), then a subset limited to restrictive lung disease (Cohort B). We further refined Cohort B by excluding patients who had volume reduction (e.g., right middle lobectomy, segmentectomy, or sub-anatomic wedge resection), creating Cohort C. Cohort C represents the most uniform population—patients with restrictive lung disease and unaltered transplanted lungs—but it is also the smallest. The CHUM Research Ethics Board approved the study (protocol #2024–11400).

The primary outcome was peak posttransplant forced expiratory volume in 1 s (FEV1). This endpoint is widely accepted in the literature as a benchmark of graft performance.^11^, ^12^, ^13^ We also evaluated the incidence of baseline lung allograft dysfunction (BLAD), defined as the failure to achieve a ‘normal’ baseline i.e., both FEV1 and forced vital capacity (FVC) to be above or equal to 80% of population-based values for at least 2 consecutive spirometry test taken 3 or more weeks apart.^14^ Secondary outcomes included overall survival, 1-year post-transplant FEV1, peak FVC, primary graft dysfunction (PGD), time to normalization (FEV1 and FVC ≥80%) and CLAD incidence.

### Statistical analysis

**Figure 2:**
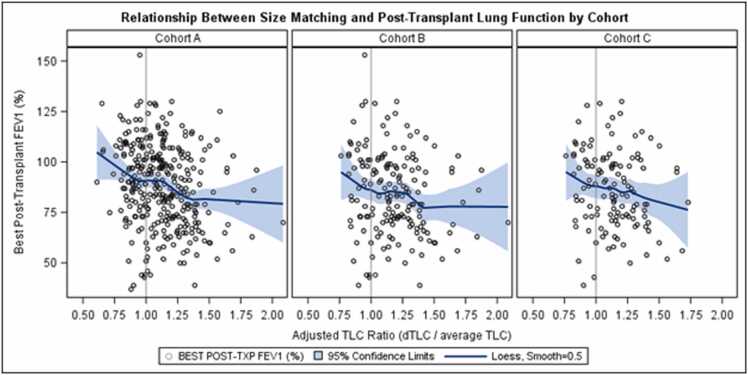
Scatter plots depicting relationship between adjusted TLC ratio and peak posttransplant FEV1 for cohorts A (all bilateral lung transplants), cohort B (restrictive lung disease) and cohort C (restrictive lung disease without intraoperative volume reduction)

Continuous variables are presented as mean ± standard deviation and categorical variables as counts (%). The relationship between adjusted TLC ratio and post-transplant lung function was explored using scatterplots of adjusted TLC ratio vs best post-transplant FEV1 (Figure 2). Adjusted TLC ratio was categorized a priori as optimal (0.9-1.0), oversized (>1.0), and undersized (<0.9). These categories were used to compare primary and secondary outcomes across cohorts. Comparisons across adjusted TLC ratio groups (optimal, oversized, undersized) were performed using 1-way ANOVA for continuous variables and chi-square tests for categorical variables.

Overall survival was evaluated using the Kaplan–Meier method, stratified by adjusted TLC ratio group within each cohort. Survival time was defined as time from transplant to death or censoring, measured in years. Patients alive at last follow-up were censored. Survival curves were compared using the log-rank test, and Kaplan–Meier estimated survival probabilities were reported at 30 days, 90 days, 1 year, and 3 years.

Multivariable Cox proportional hazards regression models were fit separately for each cohort to evaluate independent predictors of mortality. Variables with sparse data or unstable estimates were excluded from the final models to ensure robustness and interpretability of results. The final models included recipient age, donor age, adjusted TLC ratio (continuous), CMV mismatch, smoking status (ever vs never), severe pretransplant restriction, and adjusted TLC ratio group. Optimal size matching was used as the reference category. Results are reported as hazard ratios (HR) with 95% confidence intervals (CI) and corresponding *p*-values.

BLAD was evaluated using multivariable logistic regression, with separate models fit for each cohort. The outcome of interest was BLAD occurrence. Covariates included recipient age, donor age, severe PGD at 72 hours, severe pretransplant restriction, urgency status, and adjusted TLC ratio group. Due to sparse data and model instability in some cohorts, variables that were not estimable were excluded from the final reported models. Severe pretransplant restriction was defined as pretransplant FVC <50% predicted or aTLC <50% predicted.

All statistical analyses were conducted using the SPSS version 18.0.0 (SPSS Inc., Chicago, IL) and SAS (64-bit version 9.3 for Windows; SAS Institute, Cary, NC). A *p* value of <0.05 was considered statistically significant.

## Results

During the study period, 413 patients underwent lung transplantation. We excluded 37 patients who underwent single lung transplants (n = 9) or lobar transplants (n = 28), yielding cohort A (n = 376). Cohort B included patients from Cohort A with restrictive lung disease (n = 204), and Cohort C included those without intraoperative lung volume reduction (n = 150). Table 1 summarizes the demographic and donor data for all 3 cohorts.

**Table 1:** Patient Demographics, Intraoperative Data, Donor Data and Several Immediate Postoperative Outcomes

		**Cohort A (N = 376)**	**Cohort B (Restrictive lung disease) N = 204**	**Cohort C (Restrictive lung disease minus lung volume reduction) N = 150**	**All N = 413**
Sex (%)	Male	198 (52.7)	90 (44.1)	60 (40)	225(54.5)
	Female	178 (47.3)	114 (55.9)	90 (60)	188(45.5)
Mean recipient age at transplant in years (SD)		55.48 (11.86)	59.75 (7.86)	60.22 (7.56)	55.38 (12.28)
Mean BMI, kg/m2(SD)		26.26(5.9)	26.27(5.87)	25.83(6.1)	26.22(5.9)
Obesity (BMI > 30 kg/ m^2^) (%)		77(20.5)	42(20.6)	27 (18)	125 (27.6)
Bridge to Transplant (%)	None	358 (95.2)	194 (95.1)	144 (96)	387 (93.7)
	Invasive mechanical ventilation	10 (2.7)	6 (2.9)	5 (3.3)	14 (3.4)
	ECMO	8 (2.1)	4 (2.0)	1 (0.7)	12 (2.9)
Urgency (%)	Standard	222 (59)	101 (49.5)	77 (51.3)	243(58.8)
	Urgent*	154 (41)	103 (50.5)	73 (48.7)	170(41.2)
Underlying lung disease (%)	Obstructive	98 (26.1)	0	0	102 (24.7)
	Vascular	15 (4)	0	0	15(3.6)
	Suppurative	52 (52)	0	0	59(14.3)
	Restrictive	204 (54.3)	204 (100)	150 (100)	229(55.4)
	Retransplantation	7 (1.9)	0	0	8(1.9)
Mean pretransplant FEV1 % predicted (SD)		44.65 (24.38)	59.18 (19.38)	58.26 (18.19)	44.84 (23.97)
Mean pretransplant FVC % predicted (SD)		56.62 (19.25)	56.49 (19.59)	55.97 (18.22)	23.97 (55.85)
Pretransplant aTLC, L (SD)		5.12 (2.02)	3.81 (1.2)	3.90 (1.29)	4.99 (2.06)
Pretransplant pTLC, L (SD)		6.02 (1.02)	6.19 (1.02)	6.24 (0.99)	5.96 (1.05)
Mean TLC % predicted (SD)		82 (37)	58 (23)	60 (23)	81 (37)
Severe pretransplant restriction (%)		80 (22.1)	79 (40.7)	59 (40.7)	94 (23.9%
Mean difference between pTLC and aTLC, L (SD)		1.14 (2.29	2.62 (1.58)	2.54 (1.58)	1.10 (2.19)
Intraoperative data					
Lung volume reduction (%)		70 (18.6)	54 (26.5)	0	77 (18.6)
Intraoperative cardiopulmonary support (%)	None	262 (69.7)	125 (61.2)	87 (58)	271 (65.6)
	Cardiopulmonary bypass	32 (8.5)	23 (11.3)	20 (13.3)	42 (10.2)
	ECMO	82 (21.8)	56 (27.5)	45 (30)	100 (24)
Donor data					
Donor type (%)	DND	298 (79)	168 (82)	121 (81)	318 (77.0)
	DCD	65 (17)	26 (13)	22 (15)	75(18.2)
	MAID	13 (3)	10 (5)	7 (5)	14(3.4)
	Missing				6(1.5)
Mean donor age, years (SD)		46.21 (15.67)	47.12 (15.40)	45.94 (15.86)	46.29 (15.62)
Donor smoking (%)	Never	187 (49.7)	101(49.5)	80(53.3)	200(48.4)
	Current	104(28.5)	59(28.9)	38(25.3)	119(28.8)
	Former	39(10.4)	18(8.8)	11(7.3)	43(10.4)
	Unknown	42(11.4)	26(12.8)	21(14.0)	51(12.3)
Donor pTLC, L (SD)		6.08 L (1.10)	5.82 L (1.02)	5.71 L (0.93)	6.12 L (1.11)
Short-term outcomes					
PGD at 72 h (%)	< 2	285(85.3)	168(82.4)	125(83.3)	343(83.1)
	2	22(5.9)	17(8.3)	12(8.0)	27(6.5)
	3	33(8.8)	19(9.3)	13(8.7)	43(10.4)
Readmission for respiratory illness in the first year (%)	Pneumonia	47 (12.5)	28 (13.7)	19 (12.7)	49 (11.9)
	Acute rejection	3 (0.8)	2 (1.0)	2 (1.3)	3 (7.3)
	Pleural effusion	7 (1.9)	3 (1.5)	2 (1.3)	10 (2.4)
	Pulmonary embolism	7 (1.9)	6 (2.9)	5 (3.3)	8 (1.9)
	Other	6 (1.7)	3 (1.5)	3 (2)	6 (1.5)

aTLC, actual total lung capacity; BMI, body mass index; DCD, donation after circulatory death; DND, donation after neurologic death; ECMO, extracorporeal membrane oxygenation; FEV1, forced expiratory volume in 1 s; FVC, forced vital capacity; MAID, medical assistance in dying; PGD, primary graft dysfunction; pTLC, predicted total lung capacity; SD Standard deviation; TLC, total lung capacity

* Emergency status is defined somewhat subjectively, but a simple rule of thumb is (1) Patients requiring hospitalization while on the waitlist, including mechanical ventilation or ECMO. (2) Maximal domiciliary oxygen requirements (> 10 L/min by nasal cannula, or similar)

### Secondary outcomes

Based on the scatter plots, the optimal adjusted TLC ratio for size matching was 0.9 to 1.0. Patients within this range were categorized as optimally size-matched, those with ratios above 1.0 as oversized, and those with ratios below 0.9 as undersized. Table 2 shows pre-transplant pulmonary function data for these categories. As expected, preoperative pulmonary function varies significantly across adjusted TLC ratio groups in all cohorts. Undersized patients generally had higher lung volumes, while oversized patients had lower lung volumes, with optimally sized patients falling in between. These differences were consistently significant for TLC measurements. Severe pretransplant restriction was more common in oversized patients in all cohorts, but this finding reached statistical significance only in cohorts A and C. This finding is important given the association between severe restriction and reduced post-transplant survival.^8^

**Table 2:** Preoperative Pulmonary Function Data per Different Adjusted TLC Ratio Categories for all 3 Cohorts

		**Adjusted TLC ratio**			
		**Optimal (0.9-1.0)**	**Oversized (>1.0)**	**Undersized (<0.9)**	***p*-value**^a^
***COHORT A (all patients)***					
**Pretransplant FEV1 % predicted (%)**	Mean (SD)	43.50 (26.05)	46.89 (23.17)	37.67 (27.02)	0.029
**Pretransplant FVC % predicted (%)**	Mean (SD)	57.25 (23.49)	55.31 (17.55)	62.28 (20.92)	0.042
**Pretransplant actual TLC (L)**	Mean (SD)	5.60 (1.65)	4.52 (1.72)	7.00 (2.21)	<0.01
**Pretransplant aTLC % predicted (%)**	Mean (SD)	94 (30)	79 (31)	109 (32)	<0.01
**Incidence of severe pretransplant restriction (%)**	N (%)	8 (13.8)	63 (26.8)	5 (8.2)	0.02
**Difference between recipient aTLC and pTLC (L)**	Mean (SD)	0.52 (1.91)	1.34 (1.89)	-0.54 (2.03)	<0.001
**Donor pTLC (%)**	Mean (SD)	5.59 (0.91)	6.33 (1.08)	5.61 (1.08)	<0.001
***COHORT B (Restrictive lung disease only)***					
**Pretransplant FEV1 % predicted (%)**	Mean (SD)	61.52 (22.01)	58.73 (18.09)	61.33 (25.15)	0.709
**Pretransplant FVC % predicted (%)**	Mean (SD)	60.78 (22.34)	54.57 (17.47)	66.38 (26.50)	<0.001
**Pretransplant actual TLC (L)**	Mean (SD)	4.50 (1.17)	3.50 (0.94)	5.01 (1.74)	<0.001
**Preatrnsplant aTLC % predicted (%)**	Mean (SD)	69 (19)	59 (15)	77 (25)	<0.001
**Incidence of severe pretransplant restriction**	N (%)	8 (29.6)	62 (44)	5 (23.8)	0.109
**Difference between recipient aTLC and pTLC (L)**	Mean (SD)	2.08 (1.36)	2.53 (1.09)	1.52 (1.67)	<0.001
**Donor pTLC (L)**	Mean (SD)	5.26 (0.66)	6.00 (1.02)	5.01 (0.79)	<0.001
***COHORT C (Restrictive lung disease without lung volume reduction)***					
**Pretransplant FEV1 % predicted (%)**	Mean (SD)	57.00 (17.19)	57.89 (17.11)	63.20 (24.27)	0.460
**Pretransplant FVC % predicted (%)**	Mean (SD)	57.47 (14.72)	53.63 (16.34)	68.60 (25.10)	0.003
**Pretransplant actual TLC (L)**	Mean (SD)	4.72 (1.28)	3.51 (0.93)	5.12 (1.70)	<0.001
**Preatrnsplant aTLC % predicted (%)**	Mean (SD)	73 (20)	58 (15)	79 (24)	<0.001
**Incidence of severe pretransplant restriction**	N (%)	5 (26.3)	47 (46.1)	4 (20)	0.041
**Difference between recipient aTLC and pTLC (L)**	Mean (SD)	1.84 (1.39)	2.58 (1.09)	1.40 (1.63)	<0.001
**Donor pTLC (L)**	Mean (SD)	5.34 (0.73)	5.87 (0.93)	5.06 (0.78)	<0.001

aTLC, actual total lung capacity; FEV1, forced expiratory volume in 1 s; FVC, forced vital capacity; pTLC, predicted total lung capacity TLC; total lung capacity; SD, standard deviation

a Between group *p*-value for each health outcome was assessed with a 1-way ANOVA and Chi Square Test

Table 3 summarizes the impact of these categories on secondary outcomes. Across cohorts, oversized grafts were consistently associated with worse post-transplant outcomes. In Cohort A, oversized grafts demonstrated significantly lower FEV1 at 1 year compared with optimally matched grafts, with a similar trend observed for peak FEV1 that did not reach statistical significance. This was accompanied by a higher incidence of BLAD. Rates of early graft injury were also higher, including increased grade 3 PGD, although this did not reach statistical significance. Undersized grafts showed the lowest incidence of BLAD, which was significant in Cohort A. The adjusted TLC ratio did not affect time to normalization of lung function or CLAD incidence.

**Table 3:** Secondary Outcomes Based on Adjusted TLC Ratio for all 3 Cohorts

		**Adjusted TLC ratio**			
		**Optimal (0.9-1.0)**	**Oversized (>1.0)**	**Undersized (<0.9)**	***p*-value**^a^
***COHORT A (all patients)***					
**FEV1 1 year post transplant (%)**	Mean (SD)	85.63 (21.73)	78.41 (20.35)	84.81 (21.27)	0.026
**Peak FEV1 post transplant (%)**	Mean (SD)	91.98 (22.48)	86.63 (19.32)	92.58 (19.68)	0.059
**Peak FVC post transplant (%)**	Mean (SD)	87.90 (23.83)	84.22 (18.30)	89.53 (19.43)	0.141
**Peak FEV1/FVC ratio**	Mean (SD)	84 (0.08)	83 (0.09)	93 (0.11)	0.704
**Peak FEF 25-75 (%)**	Mean (SD)	123.02 (49.53)	114.29 (47.44)	123.43 (52.17)	0.306
**PGD grade 3 at 72 h**	N (%)	4 (7.5)	24 (22.9)	5 (16.1)	0.097
**Time to normalization (days)**	Mean (SD)	311.03 (479.41)	270.97 (307.88)	337.45 (294.70)	0.564
**CLAD incidence**	N (%)	10 (18.9)	46 (21.6%)	11 (21.2)	0.909
**BLAD incidence**	N (%)	23 (43.4)	106 (49.5)	14 (26.9)	<0.001
***COHORT B (Restrictive lung disease only)***					
**FEV1 1 year post transplant (%)**	Mean (SD)	84.29 (25.17)	74.73 (18.79)	79.71 (23.15)	0.092
**Peak FEV1 post transplant (%)**	Mean (SD)	89.23 (26.79)	82.75 (18.70)	87.06 (22.98)	0.286
**Peak FVC post transplant (%)**	Mean (SD)	82.95 (28.94)	80.30 (18.06)	86.82 (22.46)	0.429
**Peak FEV1/FVC ratio**	Mean (SD)	83 (0.08)	83 (0.09)	81 (0.08)	0.612
**Peal FEF 25-75 (%)**	Mean (SD)	120.16 (50.86)	110.76 (47.38)	111.88 (52.55)	0.675
**PGD grade 3 at 72 h**	N (%)	1 (3.8)	13 (10.3)	3 (17.6)	0.094
**Time to normalization (days)**	Mean (SD)	260.15 (467.62)	254.43 (341.33)	367.60 (354.08)	0.675
**CLAD incidence**	N (%)	4 (15.4)	26 (20.6)	1 (5.9)	0.300
**BLAD incidence**	N (%)	13 (50)	75 (59.5)	7 (41.2)	0.282
***COHORT C (Restrictive lung disease without lung volume reduction)***					
**FEV1 1 year post transplant (%)**	Mean (SD)	84.59 (19.38)	77.13 (18.97)	81.44 (22.74)	0.306
**Peak FEV1 post transplant (%)**	Mean (SD)	89.58 (20.36)	85.21 (18.34)	88.75 (22.61)	0.579
**Peak FVC post transplant (%)**	Mean (SD)	81.46 (25.64)	82.30 (17.67)	87.56 (22.98)	0.586
**PGD grade 3 at 72 h**	N (%)	0 (0)	6 (13.3)	2 (5.7)	0.479
**Peak FEV1/FVC ratio**	Mean (SD)	85 (0.07)	83 (0.09)	82 (0.08)	0.491
**Peak FEF 25-75 (%)**	Mean (SD)	127.39 (44.71)	115.08 (45.72)	117.25 (49.22)	0.587
**Time to normalization (days)**	Mean (SD)	322.50 (522.25)	272.14 (375.01)	367.60 (354.08)	0.784
**CLAD incidence**	N (%)	2 (10.5)	16 (18)	0 (0)	0.162
**BLAD incidence**	N (%)	9 (47.4)	49 (55.1)	6 (37.5)	0.399

BLAD, baseline lung allograft dysfunction; CLAD, chronic lung allograft dysfunction; FEV1, forced expiratory volume in 1 s; FEF 25-75, forced expiratory flow at 25%-75% of forced vital capacity; FVC, forced vital capacity; PGD, primary graft dysfunction; TLC, total lung capacity; SD, standard deviation

a Between group *p*-value for each health outcome was assessed with a 1-way ANOVA and Chi Square Test.

In Cohort B, a similar direction of effect was observed, with lower 1-year and peak FEV1 in the oversized group and higher rates of grade 3 PGD, although these differences were not statistically significant. In Cohort C, no statistically significant differences were observed; however, oversized grafts remained numerically associated with lower lung function and higher early graft dysfunction. Overall, the direction of effect was consistent across all cohorts, with oversized grafts demonstrating reduced lung function and increased early graft injury, despite variability in statistical significance.

### Survival

**Figure 3:**
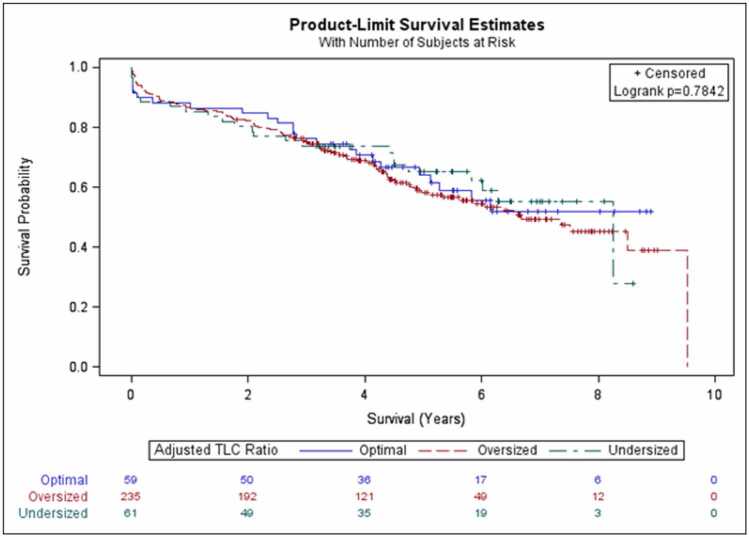
Kaplan–Meier survival curve Cohort A stratified by size-matching category

**Figure 4:**
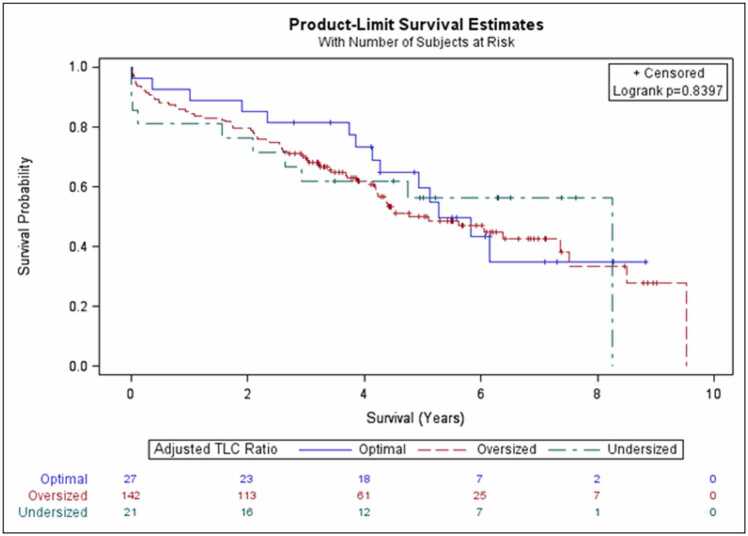
Kaplan–Meier survival curve Cohort B stratified by size-matching category

**Figure 5:**
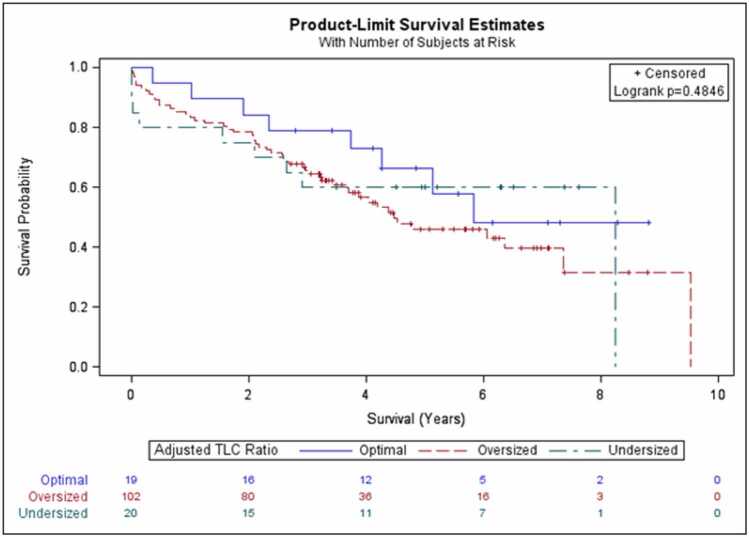
Kaplan–Meier survival curve Cohort C stratified by size-matching category

Kaplan–Meier survival analyses were performed to evaluate overall survival across categories of adjusted TLC ratio (optimal, oversized, and undersized) in Cohorts A, B, and C (Figure 3, Figure 4, Figure 5). Across all cohorts, survival curves demonstrated largely overlapping trajectories, with no statistically significant differences observed. Table 4 shows the Kaplan-Meier survival estimates at specific time-points and categorized per group per cohort.

**Table 4:** Kaplan–Meier Estimates for Overall Survival at 30 Days, 90 Days, 1 Year and 3 Years

**Adjusted TLC Ratio Group**	**30-day Survival (%)**	**90-day Survival (%)**	**1-year Survival (%)**	**3-year Survival (%)**
***COHORT A (All patients)***				
Undersized	91.8	88.5	85.2	73.8
Optimal	91.5	89.8	88.1	76.2
Oversized	95.3	91.9	86.8	75.2
***COHORT B (Restrictive lung disease only)***				
Underized	85.7	81.0	81.0	61.9
Optimal	96.3	96.3	92.6	81.5
Oversized	95.1	91.5	85.2	69.7
***COHORT C (Restrictive lung disease without lung volume reduction)***				
Undersized	85.0	80.0	80.0	60.0
Optimal	100.0	100.0	94.7	78.9
Oversized	95.1	92.2	84.3	65.6

Multivariable Cox regression analyses were performed separately for each cohort. Adjusted TLC ratio, modeled both as a continuous variable and as categorical groups, was not associated with overall survival in any cohort (Table 5). Across all 3 cohorts, size-matching (whether oversized or undersized) was not linked to survival. However, severe pretransplant restriction was consistently associated with lower survival in every cohort. Donor age was also associated with reduced survival in cohorts A and C, but this association was small, and its clinical significance is questionable.

**Table 5:** Multivariable Logistic Regression Analyses Evaluating the Association Between Adjusted TLC Ratio and Overall Survival

**Variable**	**Hazard ratio**	**95% Confidence Interval**	***p*-value**
***Cohort A (All patients)***			
Recipient age	1.011	0.994-1.028	0.19
Donor age	1.018	1.005-1.030	<0.01
Severe pretransplant restriction	1.865	1.242-2.801	<0.01
Adjusted TLC ratio (continuous)	0.697	0.198-2.454	0.57
Oversized vs optimal	1.272	0.696-2.324	0.43
Undersized vs optimal	1.006	0.522-1.941	0.98
CMV mismatch (D+/R-)	1.111	0.657-1.879	0.69
Donor smoking (yes vs no)	1.076	0.750-1.544	0.69
***Cohort B (Restrictive lung disease only)***			
Recipient age	1.012	0.981-1.044	0.45
Donor age	1.015	0.999-1.032	0.06
Severe pretransplant restriction	1.669	1.031-2.703	0.04
Adjusted TLC ratio (continuous)	0.649	0.151-2.797	0.56
Oversized vs optimal	1.395	0.634-3.067	0.40
Undersized vs optimal	1.141	0.450-2.889	0.78
CMV mismatch (D+/R-)	1.051	0.564-1.959	0.88
Donor smoking (yes vs no)	1.041	0.659-1.644	0.86
***Cohort C (Restrictive lung disease without lung volume reduction)***			
Recipient age	1.002	0.964-1.042	0.92
Donor age	1.020	1.002-1.039	0.03
Severe pretransplant restriction	2.201	1.232-3.931	<0.01
Adjusted TLC ratio (continuous)	0.449	0.064-3.150	0.42
Oversized vs optimal	1.965	0.762-5.066	0.16
Undersized vs optimal	1.273	0.430-3.769	0.66
CMV mismatch (D+/R-)	1.830	0.836-4.008	0.13
Donor smoking (yes vs no)	1.146	0.666-1.972	0.62

CMV, cytomegalovirus; TLC, total lung capacity

### BLAD

Multivariable logistic regression analyses evaluating the association between adjusted TLC ratio and BLAD were performed for each cohort (Table 6). Adjusted TLC ratio group was not significantly associated with BLAD in any cohort. In Cohort A, oversized grafts were associated with a non-significant increase in odds of BLAD, while undersized grafts were not associated with BLAD. Similar non-significant findings were observed in Cohorts B and C. In contrast, severe pretransplant restriction was consistently associated with increased odds of BLAD across all cohorts. Donor age was also associated with increased BLAD risk in Cohort A, but not in the other cohorts.

**Table 6:** Multivariable Logistic Regression Analyses Evaluating the Association Between Adjusted TLC Ratio and BLAD

**Variable**	**Odds ratio**	**95% confidence interval**	***p*-value**
***Cohort A (All patients)***			
Recipient age (per year)	0.969	0.937-1.001	0.06
Donor age (per year)	1.036	1.007-1.066	0.01
Grade 3 PGD at 72 h	1.228	0.324-4.650	0.76
Severe pretransplant restriction	4.410	1.589-12.237	<0.01
Emergency listing	1.207	0.539-2.700	0.65
Oversized vs optimal	2.246	0.707-7.136	0.17
Undersized vs optimal	0.450	0.110-1.848	0.27
***Cohort B (Restrictive lung disease only)***			
Recipient age (per year)	0.979	0.904-1.061	0.61
Donor age (per year)	1.022	0.982-1.063	0.28
Grade 3 PGD at 72 h	0.847	0.141-5.088	0.86
Severe pretransplant restriction	4.299	1.305-14.166	0.02
Emergency listing	1.698	0.537-5.368	0.37
Oversized vs optimal	1.501	0.328-6.866	0.60
Undersized vs optimal	0.774	0.101-5.932	0.81
***Cohort C (Restrictive lung disease without lung volume reduction)***			
Recipient age (per year)	0.960	0.856-1.077	0.49
Donor age (per year)	1.023	0.972-1.077	0.39
Grade 3 PGD at 72 h	0.653	0.062-6.892	0.72
Severe pretransplant restriction	5.170	1.086-24.608	0.04
Emergency listing	1.104	0.223-5.458	0.90
Oversized vs optimal	1.148	0.147-8.990	0.90
Undersized vs optimal	0.716	0.064-7.964	0.786

PGD, primary graft dysfunction

## Discussion

In this study, we demonstrated that size-matching using the adjusted TLC ratio is a feasible and clinically viable in lung transplantation. Donor-recipient pairs in the optimally sized (ratio 0.9-1.0) and undersized (0.79-0.89) groups exhibited the best long-term lung function, as measured by posttransplant FEV1. This relationship persisted both in the overall population and subsets with restrictive lung disease. Notably, oversizing was negatively associated with outcomes: recipients with an adjusted TLC ratio of greater than 1.0 experienced lower 1-year post transplant FEV1 and higher incidence of BLAD. Although the original formula proposed by Barnard et al. was intended for patients with restrictive lung disease, our findings from cohort A suggest that this approach may be applied more broadly across various lung pathologies.

Size-matching in lung transplantation remains a complex and somewhat subjective process with no universally accepted standard. Most transplant centers rely on donor and recipient height, imaging, or pTLC, and may qualitatively consider the underlying disease—preferring smaller lungs for restrictive lung disease and larger ones for obstructive disease. However, disease-specific metrics are rarely applied systematically. Barnard et al. proposed incorporating disease physiology by using the aTLC and they recommended accepting donor lungs with a pTLC within 15% to 20% above or below a calculated midpoint between recipient aTLC and pTLC—what we refer to as the adjusted TLC ratio. However, several issues arise with this approach. First, they range of 15% to 20% threshold appears arbitrary. Second, no study, to our knowledge, has reported the clinical performance of this strategy. Finally, there has never been a study comparing the adjusted TLC ratio to other size-matching strategies.

We aimed to address these gaps. First, we used scatterplot analyses to empirically define an optimal range for the adjusted TLC ratio. This approach converged on a ratio 0.9 to 1.0 as the range associated with the highest peak FEV1. Although the relationship was nonlinear and difficult to model precisely, this empirically derived range represents an improvement over the arbitrary thresholds previously proposed. While not definitive, it provides a practical guide that may be refined through larger, multicenter studies with greater statistical power and generalizability.

Second, we addressed the lack of data on the efficacy of this formula in real-world applications. Using the empirically derived thresholds, we categorized patients as optimally size matched, undersized, or oversized. One consistent finding across cohorts is that oversizing correlates with worse outcomes—including lower FEV1 and higher BLAD incidence. This finding challenges the assumption that larger donor lungs inherently provide superior function, a finding that was reported in recent publications.^1^

The physiology underlying these findings is particularly relevant in restrictive lung disease. In conditions like idiopathic pulmonary fibrosis, the chest cavity tends to stiffen and contract over time,^15^ and there is currently no reliable method to evaluate chest wall stiffness preoperatively. Conversely, obstructive lung disease results in hyperinflation.^16^ Some proponents of size matching based on ideal lung size (derived from height or pTLC) argue that the chest wall can remodel to accommodate the donor lung over time, and several studies have shown that chest cavity volumes often normalize within 1-year post-transplant, even in cases of marked chest wall distortion.^17^ This rationale has supported the practice of implanting a normal-sized lung with the expectation of postoperative remodeling. However, this becomes problematic in restrictive lung disease, where transplantation a “normal-sized” lung into a markedly contracted and noncompliant chest cavity may be physiologically untenable. Options such as delayed chest closure or prolonged ventilator weaning, or surgical lung volume reduction (e.g., wedge resection, lobectomy) are often required.^18^, ^19^ However, our data, as well as other publications, suggest that the capacity for remodeling may be limited, particularly in patients with restrictive pathology. Implanting and “ideal-sized” lung into a non-compliant chest cavity may disrupt chest wall mechanics, potentially explaining the poorer outcomes seen with oversizing in some reports—whether measured by 1-year post-transplant FEV1 or peak FEV1.^1^, ^20^, ^21^ Taken collectively, these observations reinforce that oversizing should be approached with caution. Importantly, this interpretation must be considered in the context of the underlying disease severity.

An additional notable finding is the increased incidence of severe pretransplant restriction in the oversized group. Severe restriction—defined as either an aTLC of FVC less than 50% of predicted on pretransplant pulmonary function testing—was associated with reduced long-term survival, consistent with prior literature.^3^, ^8^ Patients with severe pretransplant restriction were also more likely to receive oversized lungs, raising the question of whether poorer long-term outcomes in this group are attributable to the size-matching strategy or to the severity of the underlying disease. Beyond survival, severe pretransplant restriction was also associated with poorer long-term lung function and increased BLAD incidence. To our knowledge, this is the first study to demonstrate this association. However, our sample size is insufficient to disentangle these effects. Larger studies, ideally multicenter of prospective in design, are needed account for severe pretransplant restriction and clarify this relationship

A final barrier to adopting the adjusted TLC ratio is the lack of comparative data. Meaningful comparisons with other strategies have not been previously possible because the adjusted TLC ratio lacked both an empirically defined optimal range and clinical validation. Our study addresses these foundational gaps. However, we cannot yet recommend replacing existing size-matching strategies, direct comparative studies are required determining its relative performance.

Our study has several limitations. First, we lacked a control group using alternative size-matching strategies such as height, pTLC, or computed tomography volumetrics. We plan to address this in a future study. Second, this is a retrospective, single-center analysis and thus subject to selection bias and unmeasured confounders. Third, we excluded patients with lobar and single lung transplants, so our findings do not inform size matching in this population. Finally, although we used peak FEV1 as our primary outcome due to its availability and uniformity, other outcome measures may better capture the long-term impact of size-matching.

In conclusion, size-matching donor lungs to recipient using an adjusted TLC ratio of 0.79 to 1.0 may translate into better long-term outcomes in lung transplantation compared to ratios outside of this range. By incorporating the aTLC, this matching strategy accounts for the patient’s underlying pathology and its effect on chest wall mechanics. Oversizing, particularly in patients with restrictive lung disease may lead to worse long-term lung function. Additional studies are needed to validate this strategy in larger, multicenter groups and to compare it directly with existing size-matching strategies. Future research should also consider the frequency and impact of severe pretransplant restriction on long-term outcomes.

## Funding

No funding was received for this study.

## Declaration of competing interest

The authors declare the following financial interests/personal relationships which may be considered as potential competing interests: the only potential conflict of interest is author Dr. Matthieu Glorion’s proctoring contract with Intuitive Surgical, which is unrelated to this study. There are no other conflicts of interest to disclose. The remaining authors declare that they have no known competing financial interests or personal relationships that could have appeared to influence the work reported in this paper.
